# Efficacy of an Internet-Based Intervention to Promote a Healthy Lifestyle on the Reproductive Parameters of Overweight and Obese Women: Study Protocol for a Randomised Controlled Trial

**DOI:** 10.3390/ijerph17228312

**Published:** 2020-11-10

**Authors:** Gemma Biviá-Roig, Ruth Blasco-Sanz, Ana Boldó-Roda, M. Dolores Vara, Tamara Escrivá-Martínez, Rocío Herrero, Valentina Lucia La Rosa, Rosa M. Baños, Juan Francisco Lisón

**Affiliations:** 1 Department of Nursing and Physiotherapy, Faculty of Health Sciences, University CEU-Cardenal Herrera, CEU Universities, 46115 Valencia, Spain; 2 Department of Gynecology and Obstetricia, La Plana University Hospital, 12540 Vila-Real, Spain; ruth_blasco@hotmail.com (R.B.-S.); anaboldoroda@gmail.com (A.B.-R.); 3 Department of Personality, Evaluation and Psychological Treatment, Faculty of Psychology, University of Valencia, 46010 Valencia, Spain; m.dolores.vara@uv.es (M.D.V.); tamara.escriva@uv.es (T.E.-M.); banos@uv.es (R.M.B.); 4 Polibienestar Research Institute, Universitat de Valencia, 46022 Valencia, Spain; rocio.herrero@uv.es; 5 CIBER of Physiopathology of Obesity and Nutrition CIBERobn, CB06/03 Carlos III Health Institute, 28029 Madrid, Spain; juanfran@uchceu.es; 6 Department of Educational Sciences, University of Catania, 95124 Catania, Italy; valarosa@unict.it; 7 Department of Biomedical Sciences, Faculty of Health Sciences, University CEU-Cardenal Herrera, CEU Universities, 46115 Valencia, Spain

**Keywords:** obesity, infertility, diet, nutrition, physical activity, Internet

## Abstract

*Background:* Infertility is estimated to affect 15% of couples of reproductive age. Weight management problems (being obese or overweight) are among the problems that produce infertility, both in women seeking spontaneous pregnancy and in those undergoing assisted reproduction techniques. Over the last few decades, the prevalence of obesity has increased alarmingly in our society and is now considered one of the most important public health problems. The combination of diet and exercise to achieve weight loss are currently considered an effective intervention for the improvement of reproductive parameters in overweight or obese infertile women. In other population groups, it has been shown that Internet-based interventions are just as effective as traditional ones, and these cover a larger population with a good cost–benefit ratio. However, to the best of our knowledge, no studies so far have analysed any specific online interventions for this group of infertile women. Thus, the objective of this project will be to evaluate the effectiveness of an online program to promote a healthy lifestyle among women who are overweight or obese who also have a diagnosis of infertility and are on the waiting list for in vitro fertilisation treatment. *Methods*: This will be a randomised controlled clinical trial conducted in 94 women which will compare a self-administered Internet-based intervention promoting a healthy lifestyle in terms of diet and exercise (*n* = 47) to a control group that will receive standard medical care. The online program will comprise nine modules, will last for 3 months, and will be monitored every 3 months after the intervention until the final follow-up at 12 months. The main outcome will be the spontaneous pregnancy rate. Secondary outcomes will include changes in body composition, dietary and physical exercise habits, glycaemic profiles, lipid profiles, hormonal profiles, and patient quality of life related to their fertility problems. The data analysis will be done on an intention-to-treat basis. *Discussion*: The aim of this study is to increase our knowledge of the effectiveness of online interventions specifically adapted to infertile women who are overweight or obese in the promotion of healthy lifestyles.

## 1. Introduction

### 1.1. The Link between Overweight and Obesity and Fertility

It is estimated that infertility, defined as the inability of a couple to conceive after a year of frequent sexual intercourse, affects 15% of couples of reproductive age [1,2]. Overweight and obesity are among the different causes of infertility. A high body mass index (BMI) is related to difficulties in conceiving, both in women seeking spontaneous pregnancy and in those undergoing assisted reproduction techniques [3]. Over the last few decades, the prevalence of obesity has increased alarmingly [4] and is considered one of the most important public health problems in the Western world [5,6]. 

Some authors have observed that the probability of conception in each cycle is reduced by 8% in overweight women and 18% in obese women [7]. In addition, a high BMI is associated with menstrual irregularities [8,9] and anovulatory cycles [9,10]. Other studies indicate that obesity causes alterations in the development [10] and quality of oocytes [11,12,13] and the endometrium [14] as well as higher miscarriage rates [12]. Moreover, obese women with a diagnosis of infertility who undergo assisted reproduction treatments require higher doses of medication [15,16] and longer periods of ovarian stimulation [15]. In addition, the cancellation of cycles is higher in these women because they have a lower ovarian response [17,18,19]. Another study indicates that the chances of achieving pregnancy in an in vitro fertilisation (IVF) treatment decrease with each unit increase in BMI [20]. Specifically, these authors reported that by raising BMI by one unit, the odd for pregnancy was decreased by 0.84 (95% confidence interval 0.73–0.97).

### 1.2. Maternal–Foetal Complications Resulting from Overweight and Obesity 

Obesity not only negatively impacts fertility, but also implies a significant increase in maternal and perinatal morbidity and mortality. Maternal complications include hypertensive disorders such as pre-eclampsia, gestational diabetes, thromboembolism, instrumented vaginal deliveries, caesarean sections, surgical and anaesthetic complications, postpartum haemorrhage, infection, surgical wound dehiscence, and puerperal endometritis [21,22,23,24,25,26]. In addition, during the postpartum period, overweight mothers are more likely to suffer from hypertension and thromboembolism, which means they have a higher risk of maternal mortality [27].

Foetal risks associated with obesity include intrauterine and neonatal death, macrosomia, premature birth, congenital anomalies, and a greater number of admissions to neonatal units [28]. Likewise, the children of obese women are more likely to develop obesity, type 2 diabetes, and cardiovascular disease throughout their lives, usually at an early age [29]. Given the fertility problems that come with being overweight or obese, and the substantial risks to maternal and foetal health, it is not surprising that gynaecologists and assisted reproduction professionals warn women about the importance of optimising their BMI to improve their fertility [30,31] and reduce obstetric complications [32].

Thus, the increase in the prevalence of obesity in recent years, together with the alterations in fertility that it entails, poses a new challenge in the area of reproductive medicine. In fact, the national health system of some countries restricts assisted reproduction treatments until women have achieved sufficient weight loss [33,34]. 

### 1.3. Lifestyle and Fertility in Women Who Are Overweight or Obese

Overweight and obesity are closely linked to the development of unhealthy lifestyles, based mainly on inadequate eating habits and sedentary lifestyles [4]. In recent years, the link between lifestyle and female reproductive health has received special attention. The results of several studies indicated that some of the main factors that can negatively impact fertility are BMI, diet type, physical activity levels, stress, and consumption of toxic substances such as tobacco and alcohol [35]. Indeed, some studies showed that a modest weight loss of 5%–10% can significantly improve reproductive health [36,37]. 

In addition, weight loss through physical exercise improves metabolic function and improves both insulin resistance and female hormonal profiles [37]. The results of a recent meta-analysis show that interventions based on a combination of diet and physical activity are the most effective for weight loss and that, together, these contribute to improving reproductive parameters in obese or overweight women [38].

Given their huge potential as tools to provide these types of interventions, among others, information and communication technologies (ICTs) are now one of the main drivers of change in health systems [39]. A systematic review of Internet-based physical activity and/or nutrition programs concluded that online interventions are as effective as traditional ones [40]. In fact, there is evidence to suggest that Internet-based lifestyle modification interventions and, in particular, programs designed to help reduce excess weight and treat obesity significantly favor patient adherence to such programs [41]. In addition, Internet-based interventions can be self-administered and, because they are easy to access, they can reach wide audiences while also achieving a good cost–benefit ratio [42].

In this context, several authors have demonstrated the effectiveness of online interventions on weight loss in the general population [43,44,45,46,47]. However, despite the significant increase in ICTs in recent years, no publications in the academic literature have focused on the use of Internet-based interventions in infertile women who are overweight or obese. Thus, we propose carrying out a study to investigate whether participation in an online program designed to promote healthy lifestyles can affect the reproductive parameters of infertile women who are overweight or obese. 

## 2. Materials and Methods 

### 2.1. Study Objectives

The objective of this project is to evaluate the effectiveness of a 3-month Internet-based program focusing on the promotion of healthy lifestyles (especially weight loss and healthy eating and physical exercise habits) in women who also have a diagnosis of infertility and are on the waiting list for IVF treatment. We hypothesise that completion of this online program designed to promote healthy lifestyles by infertile obese or overweight women will improve their body composition and increase their spontaneous pregnancy rate compared to a similar group of patients receiving standard medical treatment. This study will adhere to the Consolidated Standards of Reporting Trials (CONSORT) standards for randomised trials [48] and the CONSORT-EHEALTH (Electronic and Mobile HEalth Applications and onLine TeleHealth) guidelines [49]. This protocol was based on the Standard Protocol Items: Recommendations for Interventional Trials (SPIRIT) guidelines [50] and was registered at www.ClinicalTrials.gov with reference number: NCT04275869 (Registration date: 19 February 2020).

### 2.2. Study Design, Setting, and Timing

**Figure 1:**
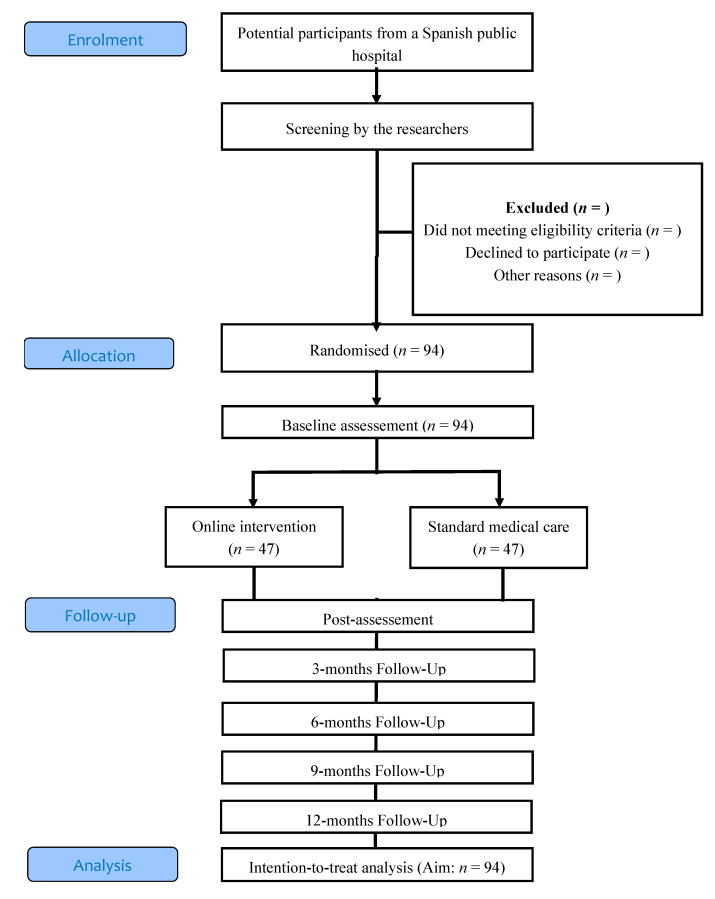
Flowchart of the study design.

A randomised controlled clinical trial will be conducted which will compare two conditions: (a) a self-administered online intervention for the promotion of a healthy lifestyle versus (b) standard medical care. The study variables will be collected at baseline, post-intervention (3 months), and then every 3 months until the start of IVF treatment. The study flowchart showing the proposed progression of the patients through the trial is shown in Figure 1.

### 2.3. Study Participants

The participants will be recruited at the Obstetrics and Gynaecology Service at the La Plana University Hospital of Villarreal, in Castellon (Spain), by the gynaecologists working there. Participants in the study must meet all of the following inclusion criteria: (1) women of childbearing age who are (2) overweight (BMI > 25 kg/m^2^) or obese (BMI > 30 kg/m^2^), (3) diagnosed with primary infertility, (4) are on the waiting list for IVF treatment, and (5) have access to the Internet. The following patients will be excluded from the study: (1) women aged over 40 years with (2) morbid or extreme obesity (BMI > 40 kg/m^2^), (3) bilateral obstruction of the fallopian tubes, (4) endometriosis, (5) amenorrhea not due to polycystic ovary syndrome, (6) patients requiring a preimplantation genetic diagnosis, (7) lonely gestational desire, (8) women whose partner has a severe male factor (oligoasthenoteratozoospermia), or (9) physical impairments precluding participation in physical activity. 

### 2.4. Sample Size and Power

We determined the sample size a priori using G-Power software (version 3.1.9.2) taking into account a probability of alpha of 0.05 and an observed power of 0.8. Based on statistics from the Spanish Fertility Society [51], we assume there will be a 20% pregnancy rate in the control group. We anticipate a 50% pregnancy rate in the intervention group based on the results from previous studies that used the pregnancy rate in previously infertile obese women as the main outcome variable after a weight loss program [52]. Thus, we determined that a total of 76 participants will be required in this study. In anticipation of possible losses, and considering descriptions in the literature of dropout rates in online interventions in this population group [53], we will increase the sample size by 25% to include a total of 94 women who will be randomly assigned to the intervention group or the control group at a 1:1 allocation ratio.

### 2.5. Randomisation and Blinding

An independent researcher will use a computerised random number generator to produce the random numbers used to allocate each participant to the intervention or control group. Given the relevance of age in female fertility and BMI (used to classify patients as overweight or obese), the sample will be stratified by age and BMI (overweight or obesity) in order to carry out the randomisation of the sample. For stratification based on age, the cut-off point will be 35 years [54]. The allocation sequence will be concealed in corresponding sequentially numbered opaque envelopes. After a participant has completed the baseline assessments, the research coordinator will open the envelope to reveal the group allocation to the participant. Participants will be informed of their group assignment at that time. The randomisation sequence will only be known by the researcher performing the randomisation and it will remain hidden from the other researchers throughout the duration of this study. This type of online interventions will not allow the allocation of masks to the gynaecologists or the participants, but the data analyst will be blinded to treatment allocation and outcomes. In any case, to avoid bias from inter-observer variability, all the measurements will be taken by the same investigator.

### 2.6. Procedure

Gynaecologists at the Hospital Reproduction Service will assess which patients meet the eligibility criteria. Women who meet these criteria will be given a document containing detailed information about the study and will be offered the possibility of voluntarily participating in it. Those who accept the invitation to participate will have to sign a written informed consent to their participation. 

Participants assigned to the control group will receive the standard treatment received by women on the waiting list for IVF treatment, according to the hospital’s protocol. This consists of regular gynaecological visits, and the Reproduction Service gynaecologists will recommend healthy lifestyle habits and give the patients a document detailing a specific diet they should follow for weight loss. Participants allocated to the intervention group will be invited to participate in an Internet-based program that promotes a healthy lifestyle based on the combination of diet and physical exercise and adapted to the specific characteristics of obese or overweight infertile women. The online platform we will use was developed by our research team based on our previous experience in online programs for promoting health in obese and overweight people [55,56,57]. This program consists of 9 modules and will last for a total of 12 weeks. The online intervention will be explained to the women and they will be given instructions about how to access the modules, use them, and complete the questionnaires. The modules will remain active until the end of the 12-month follow-up period and participants will be able to access them at any time to review their contents. The criteria for any patient to interrupt their participation the study before the end of the intervention will be their decision to: (1) start an IVF treatment at a private centre that does not require a waiting list or (2) abandon the study.

### 2.7. Online Intervention

The “Living Better” program is a computerised intervention which is self-administered through the Internet by accessing the www.psicologiaytecnologia.com website. The treatment protocol comprises 9 modules which incorporate psychological strategies to promote healthy lifestyles by gradually changing eating and physical activity habits. We will recommend the participants complete one module per week and will establish a period of 12 weeks to complete the entire program. The modules contain multimedia elements (videos, images, and texts) and will be presented sequentially so that the participants advance step by step through the program. The structure of each module is similar: first, questions related to the previous module are posed, the contents of the current module are explained, the participants are given exercises to complete, and then they will complete self-assessed test. Next, the tasks the participants must perform to practice what they learned in the module are indicated. The system allows its users to review the contents as many times as they wish.

The modules are:

M0. Welcome

The purpose of this module is to welcome the participants to the program, describe how it works and its structure, explain its main objectives and contents, and motivate the participants to start their participation in the program.

M1. Preparing to Change My Lifestyle

The objective of this module is to offer information on the importance of identifying motivations for changing one’s lifestyle. It will add more detail to the costs and benefits of maintaining or changing certain habits in terms of one’s general and reproductive health. Finally, specific and manageable objectives will be established to achieve the proposed changes.

M2. Towards a Healthy Lifestyle

The aim of this module is to reflect upon the benefits of a good diet, and to understand the role that physical activity and exercise plays in our day-to-day lives. Finally, patients will be given the key elements to start becoming more active in their everyday lives.

M3. Identifying Barriers 

The objective of this module is to identify one’s barriers to adopting the recommendations for healthy eating and increased physical activity. Different alternatives and solutions will be proposed to help patients deal with these obstacles and to overcome them. Among others, these might include advice and tricks for mealtimes, promoting mindful eating, and individually tailoring the amounts and progression of physical activities to avoid fatigue and help avoid abandonment. 

M4. Approaching My Goals

The purpose of this module is to identify the role that thoughts play in making choices about eating and physical activity habits. Strategies will be provided to help individuals become aware of thoughts they may have which could interfere with their stated goals or objectives. Specifically, the ABC technique (of irrational beliefs) will be explained and the participants will be given some tips for healthy eating and leading a more active lifestyle.

M5. Regulating My Emotions

This module will provide information about emotional eating. Participants will be helped to identify the role that food plays in the regulation of their emotions. In addition, self-control and mindfulness techniques will be provided as alternative strategies to managing their emotions.

M6. Overcoming Barriers

The objective of this module is to provide more information about the obstacles and barriers that usually appear during processes of change, and to teach the participants other coping strategies such as problem-solving techniques.

M7. Looking at Myself in the Mirror

The aim of this module is to offer users information about the impact that body image can have on well-being and health. Participants will be helped to identify concerns about their body image and work on the development of a positive body image. Finally, they will learn what assertiveness is and be taught some techniques to put it into practice.

M8. And Now What...?

The goal of this module is to summarise all the previous concepts and techniques, reinforce the changes the users may have made, and establish strategies to maintain these changes and prevent possible relapses. 

Each module will also include related content on how being overweight or obese affects fertility.

### 2.8. Interactive Elements of the “Living Better” Online Program

The web platform includes two interactive tools that will be available during the intervention: (1) an activity log which allows users to record activities related to their eating and physical activity habits; (2) a feedback section available on a weekly basis which will use graphics to help users visualise their progress in establishing new habits. In addition, the program also includes a self-evaluation protocol that will be activated before the intervention commences, at the end of each module, at the end of the intervention, and during the follow-up phase. A mobile device application used to quickly record daily eating and physical activities as well as other variables such as mood will also integrate with the online platform.

### 2.9. Support during the Intervention

The online platform will be configured to send a reminder email to the participants if they stop accessing the modules for more than two weeks. This email will aim to encourage them to continue the program and will remind the users of the importance of completing the tasks. After three weeks without accessing the platform, one of the researchers will call the participant to try to help them solve any difficulties or doubts they may have in relation to the use of the web-based system or application.

### 2.10. Covariates

The sociodemographic and anthropometric data of the participants (age, educational level, income level, occupation, etc.) will be collected at baseline. Obstetric data such as time trying to get pregnant, menstrual cycle duration, frequency of sexual intercourse, and lifestyle (hours of sleep and drug, tobacco, and alcohol use) will also be collected.

### 2.11. Primary Outcomes

The primary outcome will be the spontaneous clinical pregnancy rate (pregnancy with ultrasound visualisation of the gestational sac at week 7 of pregnancy) and evolution (pregnancy with ultrasound visualisation of the gestational sac and a heartbeat after 20 weeks of gestation [58].

### 2.12. Secondary Outcomes 

The secondary outcomes will be the following:

#### 2.12.1. Body Composition 

The following measurements will be recorded:

- The patient body mass index (BMI) will be calculated using the formula: weight (kg)/height (m^2^) using a SECA^®^ 780 (Seca, Hamburg, Germany) electronic balance scale with a mechanical telescopic stadiometer.
- The percentages of body fat and lean mass will be determined using a TANITA^®^ WB-150 MA bioimpedance scale (Biológica Tenología Médica SL. Barcelona, Spain)
- The abdominal perimeter will be measured with a tape-measure at the level of the navel (without compressing the skin tissue) and after drawing in air following one deep inhalation and exhalation. The person should stand with their feet together, arms at their sides, and abdomen relaxed [59].
- The waist–hip ratio (WHR). The perimeter of the waist at the height of the last floating rib, and the maximum perimeter of the hip at the level of the buttocks, will be measured with a tape-measure. The ratio will be calculated using the following formula: waist perimeter (cm) ÷ hip perimeter (cm).

#### 2.12.2. Diet

A dietary evaluation will be carried out using a semi-quantitative Food Frequency Questionnaire (FFQ), previously validated for the Spanish female population which includes 101 food items usually ingested on a day-to-day basis [60]. We will also use the Mediterranean Diet Adherence Screener (MEDAS) questionnaire used in the PREDIMED trial. This questionnaire has been validated for the Spanish population and assesses adherence to the Mediterranean diet, an eating pattern which has proven to be effective in the prevention and reduction of the incidence of various diseases such as cardiovascular pathologies [61], metabolic syndrome, and type 2 diabetes [62]. This questionnaire comprises 14 items, 12 of them on the frequency of food consumption and 2 on the dietary habits characteristic of the Spanish Mediterranean diet. Each item is assigned a value of 0 or 1; a score above 10 is considered representative of good adherence to the Mediterranean diet, while a score below 7 is considered indicative of low adherence pathologies [61,63].

The patients’ food-consumption styles will also be evaluated using the Dutch Eating Behaviour Questionnaire (DEBQ) validated in Spain [64]. This questionnaire provides scores for three different food consumption styles which can contribute to or mitigate the development of excess weight: “emotional eating”, “external eating”, and “restrictive eating”.

#### 2.12.3. Physical Activity

The patients’ physical activity levels will be assessed using the International Physical Activity Questionnaire Short Form (IPAQ-SF) [65]. This is a self-administered questionnaire comprising 7 items which collects information about the physical activity the survey has completed in 7 days prior to completing the test. The questionnaire also collects information about the level of sedentary behaviour, which included the number of hours they sat per day, including the time spent sitting at a desk, reading, or sitting or lying down watching television. The IPAQ-SF will be used to calculate the total number of minutes and days the person has engaged in physical activity by adding all physical activity category scores from the prior 7 days together. This data will be converted into metabolic equivalent of task minutes per week (MET-min/week), using the formula published by Ainsworth et al. [66], to classify their physical activity levels as high (>1500 MET-min/week), moderate (600–1500 MET-min/week), or low (<600 MET-min/week). The IPAQ has been validated in 12 countries [67] and has adequate psychometric properties, and the short version (the IPAQ-SF) has shown acceptable validity in an adult Spanish population [68]. 

#### 2.12.4. Lipid and Glycaemic Profiles 

A blood sample will be collected and used to determine levels of glucose, insulinemia, triglycerides, cholesterol, low-density lipoprotein, and high-density lipoprotein.

#### 2.12.5. Female Hormonal Profile 

Blood will also be drawn between the second and fourth day of each patient’s menstrual cycle in order to determine the following hormonal levels: anti-Müllerian hormone, follicle-stimulating hormone (FSH), luteinising hormone (LH), estradiol, and thyroid-stimulating hormone (TSH). 

#### 2.12.6. Ultrasound Values 

The number and size of antral follicles, endometrial morphology and thickness, and the follicular and endometrial time concordance of the menstrual cycle will be recorded by performing a vaginal ultrasound examination with the participants seated in a gynaecological chair and the examiner using a General Electric LOGIQ P3 Doppler ultrasound device. 

#### 2.12.7. Quality of Life Related to Fertility Problems 

The Fertility Quality of Life Questionnaire (FertiQol) [69] will be used to assess patient quality of life related to their fertility problems. The FertiQol comprises 24 items which test the impact of four factors related to fertility problems: emotional, mind–body, relational, and social domains. This questionnaire has been previously validated in six countries, has been translated into 20 languages (including Spanish), and shows adequate psychometric properties [69].

All the tests and questionnaires described above will be completed by each participant under the same experimental conditions in terms of the time of day (in the morning, between 8:00 and 10:00 a.m.) and test location. Table 1 shows an overview of outcomes and timings of assessments for each outcome.

### 2.13. Statistical Analysis

First, we will calculate the descriptive statistics both for the sociodemographic data and the scores obtained for the various aforementioned assessment scales. To compare the rate of spontaneous clinical pregnancy and evolution of the intervention group versus the control group, χ² tests will be performed. For the quantitative variables in the study (physical activity levels, dietary habits and food ingestion styles, body composition variables, hormonal profiles, ultrasound values, emotional well-being, and motivation) a mixed two-factor ANCOVA will be carried out, using the group as the inter-subject factor (intervention and control groups) and time as the intra-subject factor (pre-intervention, 3 months post-intervention, and 12 months after the start of the intervention). The analysis will be adjusted for age, BMI and spermatic quality. The effect size (ηp2) and the 95% confidence intervals (95% CIs) for the difference in means will be used to measure the extent of any differences. All the statistical analyses will be carried out on an intention-to-treat basis using SPSS software (version 22.0) for Windows (IBM Corp., Armonk, NY, USA).

### 2.14. Ethical Considerations

This project was approved by the Ethics Committees at the La Plana University Hospital in Villarreal and at the CEU Cardenal Herrera University in Valencia (both in Spain) and follows the fundamental principles established in the Declaration of Helsinki, the Convention on Human Rights and Biomedicine (Oviedo agreement), and the UNESCO Universal Declaration on the Human Genome and Human Rights. All the participants will be informed about the length and characteristics of the study and the voluntary nature of their participation in it. After explaining the project in detail, we will answer any questions potential participants might have about it and then they will be provided with an informed consent document that they will have to sign should they wish to participate in the study. In turn, we will provide them with the contact details for the principal investigator of the project so participants will be able to communicate with them at any time. Participants will also be informed that all the data collected during the investigation will be treated confidentially in accordance with current regulations on the protection of personal data, Organic Law 3/2018, of 5 December, on the protection of personal data and guarantee of digital rights (LOPD+GDD), and EU regulation 2016/679 of the European Parliament and Council, of 27 April 2016, regarding the protection of natural persons with regard to the processing of personal data and the free circulation of this data. The protocols we will use in this research will use standardised and validated instruments that do not pose any risks to the participants and these protocols will be implemented and supervised by expert staff. Blood collection may occasionally cause a burning feeling at the point where the needle is introduced into the skin and may cause a small bruise; more rarely it can cause transient dizziness. Ultrasound examination is a non-invasive test which cannot irradiate or affect the patient or foetus and does not replace or modify the processes involved in administering standard care practices.

## 3. Discussion

This paper outlines the protocol of a study for assessing the clinical outcomes of an Internet-based program designed to promote a healthy lifestyle in overweight or obese women with a diagnosis of infertility and who are on the waiting list for IVF treatment. We will use a randomised controlled clinical trial design to compare the effect of standard medical care versus this online intervention.

Overweight and obesity are considered to be factors that affect female fertility and decrease the probability of pregnancy by interfering with hormonal function [3,7]. In fact, although weight loss is currently recommended in Spain before starting assisted reproduction treatments, the national health systems of some countries restrict these treatments until women have achieved sufficient weight loss [31,33,34].

This group of women usually have sedentary and unhealthy habits. Previous research indicates that interventions based on a combination of diet and physical activity are the most effective for weight loss and that, together, these contribute to improving reproductive parameters in this population group [38]. However, these measures may be insufficient if patients do not receive adequate support to promote changes in their eating and activity habits or to help them learn to balance their energy consumption and expenditure [70,71]. In this context, Internet-based interventions have proven to be as effective as traditional ones [40], and even appear to show increased patient adherence to them [41].

The effectiveness of these types of online interventions for weight loss has been demonstrated for the general population in previous studies [14,38,39,40,47,65]. Despite the increasing use of Internet-based interventions for the treatment of overweight and obesity, to the best of our knowledge, no study has yet focused on this group of infertile women. Thus, from a public health perspective and given their fundamental role in treatment successes, programs specifically adapted to this population which promote healthy lifestyles and use strategies to encourage patient motivation should be developed.

This study will be carried out at the Reproduction Service in a public hospital. Among the benefits of this Internet-based intervention are: (a) it is a fully self-guided program, (b) it empowers participants by increasing their perception of self-efficacy and helps make them responsible for their own lifestyles, (c) its associated costs are low because it does not require supervision by clinical staff, (d) it reaches a high number of patients because it does not have time or geographical restrictions, (e) the progress of its participants can be monitored during the intervention without physical visits, and (f) it uses innovative ICTs to help health professionals in their work in clinical public health contexts.

Thus, participants will be able to access an online program that will help them implement lifestyle changes that could help improve their fertility (based on the two pillars indicated by the WHO: diet and physical activity) which will only require access to a computer. The possible limitations of this study are: (a) a possible sampling bias by excluding participants who do not have Internet access and (b) it is not a multicentre study because participants will only be selected from a single hospital.

## 4. Conclusions

The results of this study could lead to a cost-effective intervention that may improve the general health of this group of women, especially in terms of their reproductive parameters. Thus, this intervention could help to decrease the current socio-health costs derived from expensive assisted reproduction treatments. Similarly, this project could also pave the way for the development of subsequent research that could focus on online interventions aimed at obese or overweight men, or designed to target both relationship partners in the couple, in order to improve their reproductive health. Furthermore, the design of this novel tool specifically for this female population group could help encourage the scientific community to continue exploring the effects of interventions mediated through the Internet using online platforms.

## Figures and Tables

**Table 1:** Outcomes and timings of assessments for each outcome.

	Study Period					
	Enrolment	Allocation	Post-Allocation			
**Timepoint**	**-t1**	**Baseline**	**3 months**	**6 months**	**9 months**	**12 months**
**Enrolment:**						
Eligibility screen	X					
Informed consent	X					
Allocation		X				
**Interventions:**						
Online intervention		X	X			
Standard medical care		X	X			
**Assessments:**						
Demographics/anthropometrics		X				
Obstetric data		X	X	X	X	X
Clinical pregnancy						
Body composition (BMI, % body fat and lean mass, abdominal perimeter, WHR)		X	X	X	X	X
Diet (FFQ, MEDAS, DEBQ)		X	X	X	X	X
Physical activity (IPAQ-SF)		X	X	X	X	X
Lipid/ glycaemic profile		X	X	X	X	X
Hormonal profile		X	X	X	X	X
Ultrasound values		X	X	X	X	X
Quality of life (FertiQol)		X	X	X	X	X

BMI, body mass index; WHR, waist–hip ratio; FFQ, Food Frequency Questionnaire; MEDAS, Mediterranean Diet Adherence Screener questionnaire; DEBQ, Dutch Eating Behaviour Questionnaire; IPAQ-SF, International Physical Activity Questionnaire Short Form; FertiQol, Fertility Quality of Life Questionnaire.
